# Peripheral Inflammation Profile of Cerebellar Ataxia

**DOI:** 10.2174/011570159X379620250225075810

**Published:** 2025-02-28

**Authors:** Cuiling Tang, Qi Deng, Xinrong Yuan, Ziyan Ding, Jian Hu, Linliu Peng, Hongyu Yuan, Na Wan, Yiqing Gong, Siyu Ding, Yan Tan, Lijing Lei, Linlin Wan, Rong Qiu, Beisha Tang, Zhao Chen, Hong Jiang

**Affiliations:** 1 Department of Neurology, Xiangya Hospital, Central South University, Changsha, 410008, China;; 2 Key Laboratory of Hunan Province in Neurodegenerative Disorders, Central South University, Changsha, 410008, China;; 3 National Clinical Research Center for Geriatric Disorders, Xiangya Hospital, Central South University, Changsha 410008, China;; 4 Department of Radiology, Xiangya Hospital, Central South University, Changsha, 410008, China;; 5 National International Collaborative Research Center for Medical Metabolomics, Central South University, Changsha, 410008, China;; 6 School of Computer Science and Engineering, Central South University, Changsha, 410083, China;; 7 Hunan International Scientific and Technological Cooperation Base of Neurodegenerative and Neurogenetic Diseases, Changsha, 410008, China;; 8 Department of Neurology, The Third Xiangya Hospital, Central South University, Changsha, 410013, China;; 9 Furong Laboratory, Central South University, Changsha, Hunan, 410008, China;; 10 Brain Research Center, Central South University, Changsha, Hunan, 410008, China

**Keywords:** Cerebellar ataxia, biomarkers, blood routine examination, peripheral inflammation, receiver operating characteristic (ROC), RNA

## Abstract

**Objectives:**

The objective of this study is to determine the characteristics of peripheral inflammatory profiles and their correlations with the clinical features in patients with cerebellar ataxia.

**Methods:**

We conducted a cross-sectional study on a cohort of 140 cerebellar ataxia patients, including 74 patients with spinocerebellar ataxia (SCA), 66 patients with multiple system atrophy with predominant cerebellar ataxia (MSA-C), and 145 healthy controls (HCs). Inflammatory profiles (PLT, MPV, NLR, PLR, MLR, SII, AISI and ESR) were measured in peripheral blood, and were compared by ANOVA and Kruskal-Wallis test. The receiver operating characteristic (ROC) curve and the area under curve (AUC) were performed to determine the sensitivity and specificity of the inflammatory markers. Spearman correlation and partial correlation analysis were performed to detect the association between inflammatory profiles and clinical scales in cerebellar ataxia.

**Results:**

Inflammatory profiles from peripheral blood showed significant difference between different groups. Significant variations were observed in MPV, NLR, MLR, SII, AISI and ESR between cerebellar ataxia and HCs groups (*p*<0.05). NLR and ESR in both SCA and MSA-C groups were increased compared with HCs (*p*<0.05). The difference of MHR between SCA and MSA-C groups was observed based on HDL variation (*p*<0.05). The combination of ESR and PLT distinguished SCA from MSA-C (AUC=0.800). In addition, MLR was significantly corelated with clinical scales, including SARA and ICARS in SCA group as well as UMSARS and FAB in MSA-C group (r>0.3/r<-0.3).

**Conclusion:**

Significant variation in peripheral inflammatory profiles was firstly identified in Chinese genetic ataxias and non-genetic cerebellar ataxia cohort, which showed the potential clinical correlations between peripheral inflammatory phenotype and severity of ataxia.

## 1 INTRODUCTION

Cerebellar ataxia is a diverse collection of diseases that show similar main symptoms-gait and balance abnormalities, eye movement abnormalities, speech abnormalities and other symptoms [1]. Although the progressive rate of each type of cerebellar ataxia is different, these diseases will finally worsen quality of life and shorten life expectancy. Causes of cerebellar ataxia can be separated into genetic ataxias and non-genetic diseases [2]. Spinocerebellar ataxia (SCA) is a typical disease of genetic cerebellar ataxia, while multiple system atrophy with predominant cerebellar ataxia (MSA-C) is a characteristic part in non-genetic cerebellar ataxia [3, 4]. Compared with MSA-C, SCA showed a longer disease progression and incidences at younger age. The pathophysiology of these diseases varies due to different pathological and molecular mechanisms [5-7]. The pathology of SCAs ranged from pure cerebellum atrophy to broader neurodegeneration, including spinal cord and peripheral nerves, due to the different causative genes [3, 8-12]. In SCA, polyglutamine expansion, RNA toxicity, channel dysfunction and other molecular mechanisms were reported as interrelated pathophysiological mechanisms [13-18]. In MSA-C, obvious atrophy in the midbrain, cerebellum, brainstem and basal ganglia has been reported [4, 19, 20], which is characterized by cytoplasmic α-synuclein inclusions in oligodendrocytes [21-24].

Recently, neuroinflammation has been found to be an important pathological process in neurodegenerative diseases [25-29], including Alzheimer’s disease (AD) [30-32], Parkinson’s disease (PD) [33], SCA [34] and MSA [35-37]. Evidence showed that there was a crosstalk between central and peripheral immune cells [38-40]. In spinocerebellar ataxia type 2 (SCA2), peripheral inflammatory mechanisms may occur before the onset of ataxia [41], and previous investigations showed C3, C4, and IgG correlation with disease severity in MSA [42, 43]. Thus, neuroinflammation may be indirectly reflected through blood inflammatory biomarkers. Specifically, Neutrophil-to-lymphocyte ratio (NLR) [44, 45], platelet-to-lymphocyte ratio (PLR) [46, 47], monocyte-to-lymphocyte ratio (MLR) [48-50], systemic inflammation index (SII) [51, 52], aggregate index of systemic inflammation (AISI) [53], monocyte to high density of lipoprotein (HDL) ratio (MHR) [54, 55] and neutrophil to HDL ratio (NHR) [56, 57] were regarded as systemic inflammation indices. However, these peripheral inflammatory profiles were not systematically assessed in cerebellar ataxia.

Here, we performed a retrospective study to characterize the peripheral inflammatory profiles in 74 SCA subjects and 66 MSA-C subjects *versus* healthy controls using blood cell count, high density of lipoprotein (HDL)-derived inflammatory indices and erythrocyte sedimentation rate (ESR). We further explored the relationship between peripheral inflammation and cerebellar ataxia with various disease causes and severity.

## 2 MATERIALS AND METHODS

### 2.1 Sample Size

G*Power 3.1 program (Heinrich-Heine-Universität Düsseldorf, Düsseldorf, Germany) was used to calculate the sample size. The sample size of 159 (53 per group) was calculated for α error fixed at 5% and β error fixed at 20%, with a medium effect size (0.25) and 0.8 for power [58-60]. The sample size was calculated based on a previous study [41] that assessed the relationship between peripheral inflammation markers and clinical features in the SCA2 (significance level: 95% [α = 0.0^5^]; power: 80% [β = 0.^20^]), suggesting 44 participants. Considering a 10% exclusion rate, at least 60 patients in each group should be included.

### 2.2 Patients and Data Collection

A retrospective review of the medical records of patients diagnosed with cerebellar ataxia (n=144) at Xiangya Hospital was carried out. SCA patients (n=78) were genotyped by capillary electrophoresis of *ATXN1, ATXN2, ATXN3* and *TBP. MSA-C* patients (n=66) were diagnosed according to 2022 guidelines [61]. The exclusion criteria were as follows: 1) complicated with severe or acute infections (n=3); 2) confirmed systemic inflammatory disease (n=1); 3) confirmed hematologic or neoplastic diseases; 4) severe liver and kidney damages. Finally, 74 SCA patients and 66 MSA-C patients were included. Sex-matched healthy individuals were included as controls.

We collected demographic and laboratory characteristics for all patients. Clinical information, including the age, gender, disease duration and results of physical examination, was obtained from the archive in the hospital. SCA individuals underwent the Scale for the Assessment and Rating of Ataxia (SARA) [62, 63], the International Cooperative Ataxia Rating Scale (ICARS) [64], Barthel Index (BI) [65] and Mini-Mental State Examination (MMSE) [66]. MSA-C patients underwent the Unified Multiple System Atrophy Rating Scale (UMSARS) [67], the Scales for Outcomes in PD-Autonomic (SCOPA-AUT) [68, 69], Wexner [70], MMSE and Frontal Assessment Battery (FAB) [71]. Clinical assessments were conducted by two experienced neurologists. All study participants had clinical blood cell count, HDL, and erythrocyte sedimentation rate (ESR). The neutrophil (NEU)-to-lymphocyte (LYM) ratio (NLR; NEU/LYM), platelet (PLT)-to-lymphocyte ratio (PLR; PLT/LYM), monocyte (MON)-to-lymphocyte ratio (MLR; MON/LYM), Systemic Inflammation Index (SII; PLT×NEU/LYM), Aggregate Index of Systemic Inflammation (AISI; NEU×PLT×MON/LYM), neutrophil to high-density lipoprotein ratio (NEU/HDL; NHR), and monocyte to high-density lipoprotein ratio (MON/HDL, MHR) were calculated.

### 2.3 Statistical Analyses

SPSS 26.0 software (Chicago, IL, USA) and G*Power 3.1 program (Heinrich-Heine-Universität Düsseldorf, Düsseldorf, Germany) were used for analyses. Data were presented as numbers (%), mean (standard deviation), or median (interquartile range [IQR]) as appropriate. Differences among the three groups were assessed using ANOVA and Kruskal-Wallis test. *P* values of the posterior comparisons were adjusted by Bonferroni correction. As several markers (PLT, NLR, PLR, MLR, AISI and ESR) were correlated with ages, analysis of covariance (ANCOVA) was used to compare these markers between different groups. For comparison between the two groups, an independent sample t-test was conducted for comparison of the binary with normal distribution, and the Mann-Whitney U test was used for comparison of the binary groups with non-normal distribution variables. Chi-square tests were used to determine the difference in sex composition between the two groups. To attach the predicted probability of combined markers, logistic regression was performed. The receiver operating characteristic (ROC) curve and the area under the curve (AUC) were performed to determine the sensitivity and specificity of the inflammatory markers. Pearson or Spearman correlation test was used to assess the correlation between two variables obeying normal distribution or not. Partial correlation analysis was used to investigate the association between inflammatory markers and severity of ataxia. Missing data were removed in pairs. The differences were significant when *p*<0.05.

## 3 RESULTS

### 3.1 Demographic Characteristics of Cerebellar Ataxia Participants

Among the 140 patients diagnosed with cerebellar ataxia, 74 patients were diagnosed as SCA, including SCA1 (n=1), SCA2(n=6), SCA3(n=64), SCA6 (n=1) and SCA17 (n=2), while 66 patients were diagnosed as clinically possible or probable MSA-C. The age of MSA-C patients was older than SCA patients. Baseline clinical characteristics of patients with cerebellar ataxia are presented in Table **1**.

### 3.2 NLR and ESR of Both SCAs and MSA-C Patients were Increased

Most of the inflammatory profiles presented an abnormal distribution. MPV (*p*=0.001), NLR (*p*<0.001), MLR (*p*=0.019), SII (*p*=0.003), AISI (*p*=0.003) and ESR (*p*=0.001) in cerebellar ataxia group were found significantly increased compared with HCs group (Fig. **S1**, Table **S1**).

**Fig. (1):**
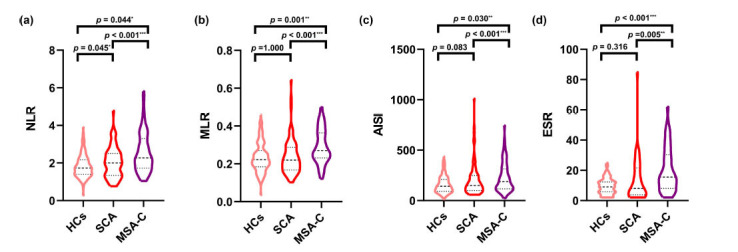
Altered peripheral inflammatory in HCs, SCAs and MSA-C subjects. The figures shows (**A**) NLR, (**B**) MLR, (**C**) AISI and (**D**) ESR levels in all three groupsparticipants: HCs, SCA and MSA-C. Variables were compared among three groups by one-way ANOVA for normally distributed data or the Kruskal-Wallis test for abnormally distributed data. *P* values of the posterior comparisons were adjusted by Bonferroni correction. **Abbreviations:** HCs, healthy controls, NLR, neutrophils-to-lymphocyte ratio, MLR, monocytes-to-lymphocyte ratio, AISI, aggregate Index of Systemic Inflammation, ESR, erythrocyte sedimentation rate, SCA, spinocerebellar ataxia, MSA-C, multiple system atrophy with predominant cerebellar ataxia. *p<0.05; **p<0.01; ***p<0.001 (Bonferroni corrected).

In subgroup analysis, age was considered as a confounder in profiles of PLT, PLR, and ESR due to age differences among SCA, MSA-C group and HCs. NLR increased in MSA-C and SCA groups compared with HCs group, with a higher level in the latter group (*p*<0.05) (Fig. **1a**). MLR, AISI, SII and ESR in MSA-C group increased compared with HCs and SCA group (*p*<0.05), while a difference was not found between the latter two groups (Figs. **1b**-**d**, Fig. **S2**). The NHR in MSA-C group was found significant increased compared with SCA because of the lower levels of HDL (Table **1**, Fig. **S2**). Correlation analysis showed HDL was unrelated to inflammatory profiles except NHR and MHR (Table **S2**). The comparative analysis between SCA3 and HCs group was conducted due to high frequency of SCA3, and the results were similar to the comparison between SCA and HCs group (Fig. **S3**, Table **S3**).

To fully eliminate the impact of age, ROC curves of SCA/MSA-C and sex-and-age matched healthy controls were examined. The AUC of individual inflammatory markers yielded from 0.593 to 0.706, whereas the AUC of combined inflammatory markers yielded from 0.680 to 0.800 (Fig. **S4**).

### 3.3 Inflammatory Profiles were Correlated with the Severity of Cerebellar Ataxia

**Fig. (2):**
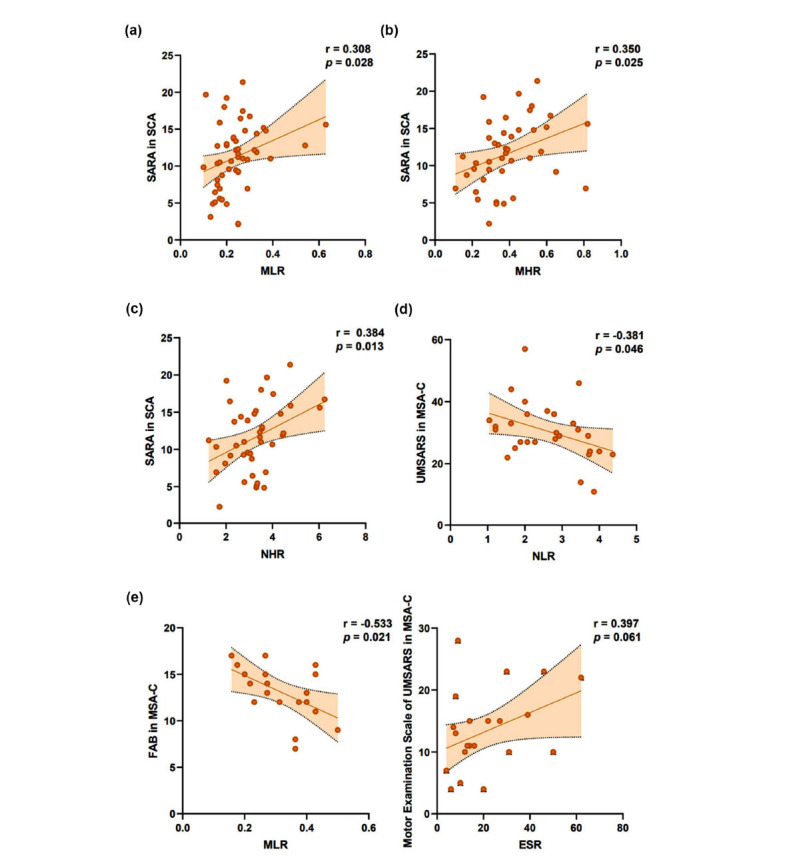
Correlation analysis between inflammatory markers and the severity of ataxia was presented. (**A**) MLR, (**B**) MHR and (**C**) NHR were positively correlated with SARA total score in SCA group. In MSA-C group, (**D**) NLR, (**E**) MHR and (**F**) were correlated with severity of disease. All SCAs patients carried out SARA, ICARS, BI and MMSE (n=52) and MSA-C patients carried out UMSARS, MMSE, SCOPA-AUT, Wexner, and FAB (n=28) were included. R values and *p* values calculated using Pearson correlation or Spearman correlation coefficient analysis are indicated on each dot plot. After Bonferroni correction, adjusted *p* values were non-significant. **Abbreviations:** MLR, monocytes-to-lymphocyte ratio, MHR, monocyte to high-density lipoprotein ratio. NHR, neutrophil to high-density lipoprotein ratio, NLR, neutrophils-to-lymphocyte ratio, ESR, erythrocyte sedimentation rate, SARA, the Scale for the Assessment and Rating of Ataxia. UMSARS, the Unified Multiple System Atrophy Rating Scale, FAB, Frontal Assessment Battery, SCA, spinocerebellar ataxia, MSA-C, multiple system atrophy with predominant cerebellar ataxia.

The correlation between inflammatory profiles and severity of ataxia was evaluated. Bivariate correlations analysis found age, age of onset, and disease duration were correlative with SARA total score and ICARS subitems in SCA group. After adjusting for confounders, MLR, AISI and MHR were positively correlated with the SARA total score (r_MLR_=0.308, r_AISI_=0.278, r_MHR_=0.350, *p*<0.05) and ICARS total score (r_MLR_=0.315, r_AISI_ =0.279, r_MHR_=0.390, *p*<0.05) (Figs. **2a**-**b**, Fig. **S5**). NHR was positively correlated with SARA total score (r=0.384, *p*=0.013) (Fig. **2c**) but not with ICARS (r=0.297, *p*=0.059) (Fig. **S5**). PLR and ESR were not significantly correlated with the total score and the subitems of SARA and ICARS (Fig. **S5**). These inflammatory profiles were not correlated with the MMSE and BI scores (Fig. **S6**). In SCA3 patients, consistent results were found except MLR. MLR was not significantly correlated with the total scores and subitems of the SARA and the ICARS except the posture and gait scores (r=0.320, *p*=0.041) of the ICARS (Fig. **S7**). After Bonferroni correction, adjusted *p* values were non-significant.

In MSA-C patients, bivariate correlations analysis found age, age of onset, and disease duration were correlative with the UMSARS subitems. After adjusting for confounders, inflammatory profiles were not significantly correlated with the total score of UMSARS except NLR (r=-0.381, *p*=0.046) (Fig. **2d**, Fig. **S8**). MLR (r=-0.533, *p*=0.021) and SII (r=-0.525, *p*=0.019) were negatively correlated with the FAB score (Fig. **2e**, Fig. **S8**). These inflammatory profiles were not correlated with the SCOPA-AUT and Wexner scores except MHR, which was positively correlated with the Wexner score (r=0.498, *p*=0.030) (Fig. **S8**). After Bonferroni correction, adjusted *p* values were non-significant.

**Fig. (3):**
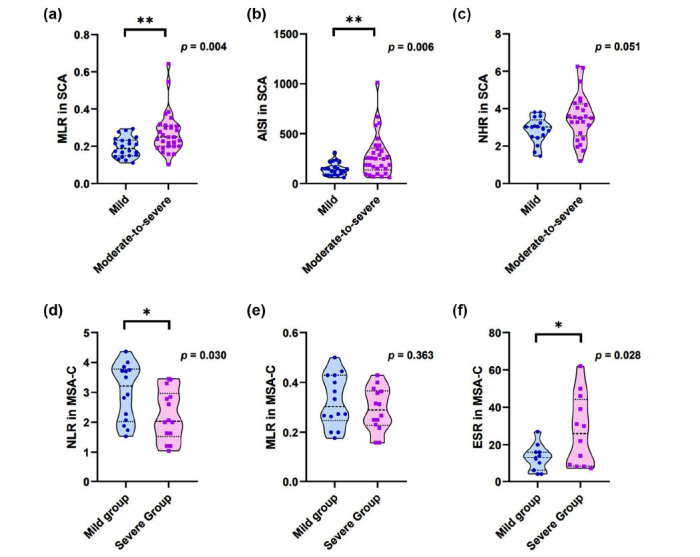
Comparisons of peripheral inflammation in different groups of SCAs and MSA-C patients. Higher levels of (**A**) MLR and (**B**) AISI were associated with moderateto-severe SCA groups, and (**C**) NHR showed an increasing trend. In MSA-C group, (**D**) NLR and (**E**) MLR showed no difference in these groups while (**F**) ESR increased in the severe groups. SCAs patients were divided into mild group (SARA <10, n=22) and moderate-to-severe group (SARA ≥10, n=30). MSA-C patients were divided into the mild group (UMSARS <29.5, n=14) and severe group (UMSARS>29.5, n=14). Variables were compared by independent sample t-test for normally distributed data or Mann-Whitney U test for abnormally distributed data. **Abbreviations:** MLR, monocytes-to-lymphocyte ratio, AISI, aggregate Index of Systemic Inflammation, NHR, neutrophil to high-density lipoprotein ratio, NLR, neutrophils-to-lymphocyte ratio, ESR, erythrocyte sedimentation rate, SCA, spinocerebellar ataxia, MSA-C, multiple system atrophy with predominant cerebellar ataxia.

In order to further clarify the correlation between the peripheral inflammatory profiles and the severity of the disease, the SCA participants were divided into mild (SARA<10) and moderate-to-severe groups (SARA≥10), while the MSA-C participants were divided into mild (UMSARS<29.5) and moderate groups (UMSARS≥29.5). We found higher levels of MLR (*p*=0.004), AISI (*p*=0.006) and SII (*p*=0.023) were associated with moderate-to-severe SCA groups (Figs. **3a**-**b**, Table **S4**). NHR in moderate-to-severe group showed an increasing trend (*p*=0.051) (Fig. **3c**). In MSA-C group, NLR (*p*=0.030) and ESR (*p*=0.028) increased in the severe group, which was consistent with the results of correlation (Figs. **3d**, **f**). Other peripheral inflammatory profiles showed no difference in these groups (Fig. **3e**, Tables **S4** and **S5**).

## 4 DISCUSSION

Cerebellar ataxia is a group of diseases with gait and balance disorders due to different causes. Easy-attached biomarkers need to be explored to trace disease progression and help to improve differential diagnosis. Our study included genetic cerebellar ataxia patients and non-genetic cerebellar ataxia patients and explored their differences in inflammatory indicators. The study showed that NLR, MLR, SII, AISI and ESR of cerebellar ataxia patients increased, indicating that peripheral inflammatory markers could potentially distinguish these patients from healthy individuals. Most of these inflammatory indicators were correlated with the severity of ataxia.

Compared with HCs, we found that NLR and ESR significantly increased in SCA and showed a higher trend in MSA group. Additionally, MLR, SII and AISI significantly increased solely in MSA-C group. Although the AUC of mono-indicator for disease discrimination was limited, the combined inflammatory indicators might achieve better performance. In addition, we observed MLR, AISI, MHR and NHR in inflammatory profiles were positively correlated with disease severity in SCA group, while NLR, MLR and SII negatively correlated with disease severity except MHR and ESR in MSA-C group. Although the correlations were no longer significant after Bonferroni correction, their correlation coefficient might still demonstrate clinically meaningful effect sizes, especially for those r>0.300 and r<-0.300.

Overall, the correlation between inflammatory profiles and clinical characteristics in MSA-C patients diverged from SCAs patients. Different pathophysiologies of MSA-C and SCA might contribute to these differences. Although neuroinflammation existed in cerebellar ataxia as mentioned above, in MSA, hypothesis was proposed that the accumulation of α-synuclein in oligodendrocytes induces microglial activation, pointing to the oligodendrocyte-neuron crosstalk [72-74], while in SCAs neuroinflammation might be triggered as a secondary response to toxicity of aggregate formation induced by polyglutamine tract in neuron [3]. Here we propose that the oligodendrocyte depletion with the progression of MSA contributes to the reduction of neuroinflammation. On the contrary, the neuron injuries induced the activation of microglial and increased the production of pro-inflammatory cytokines.

Notably, our study first identified the difference in NHR (NEN/HDL) between the SCA and MSA-C groups due to the difference in HDL levels. Previous studies have suggested that HDL has anti-inflammatory and anti-oxidative activities [75]. The decrease in HDL was regarded as the marker of peripheral inflammatory activation [76, 77]. Previous studies found a lower level of HDL in MSA patients compared with HCs, which might not deteriorate or improve the progression of MSA [78]. However, our research showed that compared with SCA patients, HDL levels were decreased in patients with MSA-C, and MHR was positively correlated with disease severity in both SCA and MSA-C groups, suggesting that low HDL underpinning high NHR might contribute to different progression rates in SCA and MSA-C. Also, our study showed that HDL was uncorrelated with peripheral inflammatory markers except NHR and MHR, indicating the decline in HDL couldn’t contribute to the increase in other inflammatory markers. Thus, HDL may independently contribute to peripheral inflammation and, therefore, involves distinct phenotypic characteristics compared to other inflammatory markers. Taken together, as MSA-C patients progress with a more rapid progression, whether peripheral inflammation and HDL levels contribute to the different rates of disease progression in SCA and MSA-C patients and what role they play needs to be further investigated.

## 5 LIMITATIONS

As for limitations, our research was a retrospective single-center study and lacked long-term follow-up visit; whether these inflammatory markers vary with disease progression remains unclear. A multi-center follow-up study across different cohorts in the future is needed. Also, our sample capacity was limited because of the outbreak of COVID-19. The serum concentrations of the C4 and C3c complement were excluded as these profiles were measured only in several patients. However, we included MHR and NHR and found their potential clinical correlation with the severity of ataxia.

## CONCLUSION

Our research identified the inflammatory profiles based on cell count as biomarkers that could indicate the severity of ataxia and help separate different types of ataxias. We first identified the difference in NHR between the SCA and MSA-C groups due to the difference in HDL levels. Furthermore, although it is necessary to expand the long-term follow-up study, it could help to explore the neuroinflammatory mechanism, thus extending disease-modifying intervention options.

## Figures and Tables

**Table 1:** Demographic features for all participants in retrospective study.

**Sex**	**HCs**	**Cerebellar Ataxias**		**Value**	**Sig.**
	**n=145**	**SCAs**	**MSA-C**		
		**n=74**	**n=66**		
Male	89	41	39	0.360	0.698
Female	56	33	27		
Average Age	47.00 (20.00)	36.96 (17.00)	52.00 (7.63)	58.600	0.000^***^
PLT (×109/L)	207.00 (57.00)	219.00 (87.00)	192.66 (44.531)	1.186	0.307
MPV (fl)	9.010 (1.420)	9.755 (1.570)	9.496 (1.696)	6.546	0.002^**^
NLR	1.733 (0.767)	2.038 (0.816)	2.270 (1.574)	19.130	0.000^***^
PLR	122.72 (39.37)	120.00 (63.07)	130.25 (43.95)	1.617	0.200
MLR	0.222 (0.085)	0.216 (0.117)	0.270 (0.133)	14.700	0.000^***^
SII	364.50 (208.76)	429.79 (167.95)	451.53 (330.83)	7.395	0.006^**^
AISI	141.47 (117.77)	149.52 (147.30)	188.33 (197.76)	8.271	0.000^***^
ESR^a^ (mm/h)	9.00 (7.00)	8.00 (17.00)	15.50 (22.00)	16.406	0.000^***^
HDL^b^ (mmol/L)	-	1.18(0.27)	1.01(0.25)	2.222	0.028^*^
NHR	-	3.147 (1.144)	3.631 (1.407)	-1.793	0.044^*^
MHR	-	0.355 (0.183)	0.386 (0.223)	-2.113	0.107

**Note:** One-way ANOVA and Kruskal-Wallis test were used to determine the distributions of sex, average age, MPV, SII, NHR and MHR. Correlation analysis found PLT, NLR, PLR, MLR, AISI and ESR were related to ages, and ANCOVA was used to compare these markers between different groups. Independent sample t-test was conducted for comparison of HDL. a. 102 HCs and 96 cerebellar ataxias patients (including 46 SCAs patients and 50 MSA-C patients) underwent ESR. b. 61 SCAs patients and 55 MSA-C patients underwent HDL. PLT, platelet. MPV, mean platelet volume. NLR, neutrophils-to-lymphocyte ratio. PLR, platelet-to-lymphocytes ratio. MLR, monocytes-to-lymphocyte ratio. SII, systemic inflammation index. AISI, aggregate index of systemic inflammation. ESR, erythrocyte sedimentation rate. HDL, high density of lipoprotein. MHR, monocyte to high-density lipoprotein ratio. NHR, neutrophil to high-density lipoprotein ratio. * *p*<0.05; ** *p*<0.01; *** *p*<0.001.

## Data Availability

The data that support the findings of this study are available from the corresponding author upon reasonable request.

## LIST OF ABBREVIATIONS

AD: Alzheimer Disease
AISI: Aggregate Index of Systemic Inflammation
AUC: The Area Under Curve
BI: Barthel Index
ESR: Erythrocyte Sedimentation Rate
FAB: Frontal Assessment Battery
HCs: Healthy Controls
HDL: High Density of Lipoprotein
ICARS: International Cooperative Ataxia Rating Scale
LYM: Lymphocyte
MHR: Monocyte to High Density of Lipoprotein Ratio
MLR: Monocyte-to-lymphocyte Ratio
MMSE: Mini-Mental State Examination
MON: Monocyte
MPV: Mean Platelet Volume
MSA: Multiple System Atrophy
MSA-C: Multiple System Atrophy with Predominant Cerebellar Ataxia
NEU: Neutrophil
NHR: Neutrophil to High Density of Lipoprotein Ratio
NLR: Neutrophil-to-lymphocyte Ratio
PD: Parkinson’s Disease
PLR: Platelet-to-lymphocyte Ratio
PLT: Platelet
ROC: Receiver Operating Characteristic
SARA: Scale for the Assessment and Rating of Ataxia
SCA: Spinocerebellar Ataxia
SCA2: Spinocerebellar Ataxia Type 2
SCOPA-AUT: Scales for Outcomes in PD-Autonomic
SII: Systemic Inflammation Index
TBP: TATA Box-binding Protein
UMSARS: Unified Multiple System Atrophy Rating Scale

## STANDARDS OF REPORTING

STROBE guidelines were followed.
