# Supplementary material for: Peripheral Inflammation Profile of Cerebellar Ataxia
Source: Curr Neuropharmacol. 2025 Feb 28;23(10):1276–86. doi: 10.2174/011570159X379620250225075810 (PMC12307985; doi:10.2174/011570159X379620250225075810)
Supplement: Supplementary file 1 [file CN-23-10-1276_SD1.pdf]

Supplementary Material

Peripheral Inflammation Profile of Cerebellar Ataxia

Cuiling Tang[1], Qi Deng[1], Xinrong Yuan[1], Ziyan Ding[1], Jian Hu[1], Linliu Peng[1], Hongyu Yuan[1], Na Wan[1], Yiqing Gong[1], Siyu Ding[1], Yan Tan[1], Lijing Lei[1], Linlin Wan[1,2,3,4,5], Rong Qiu[6], Beisha Tang[1,2,3,7], Zhao Chen[1,2,3,7,*] and Hong Jiang[1,2,3,5,7,8,9,10,*]

[1]Department of Neurology, Xiangya Hospital, Central South University, Changsha, 410008, China; [2]Key Laboratory of Hunan Province in Neurodegenerative Disorders, Central South University, Changsha, 410008, China; [3]National Clinical Research Center for Geriatric Disorders, Xiangya Hospital, Central South University, Changsha 410008, China; [4]Department of Radiology, Xiangya Hospital, Central South University, Changsha, 410008, China; [5]National International Collaborative Research Center for Medical Metabolomics, Central South University, Changsha, 410008, China; [6]School of Computer Science and Engineering, Central South University, Changsha, 410083, China; [7]Hunan International Scientific and Technological Cooperation Base of Neurodegenerative and Neurogenetic Diseases, Changsha, 410008, China; [8]Department of Neurology, The Third Xiangya Hospital, Central South University, Changsha, 410013, China; [9]Furong Laboratory, Central South University, Changsha, Hunan, 410008, China; [10]Brain Research Center, Central South University, Changsha, Hunan, 410008, China

1875-6190/25 $65.00+.00 **© 2025 Bentham Science Publishers**

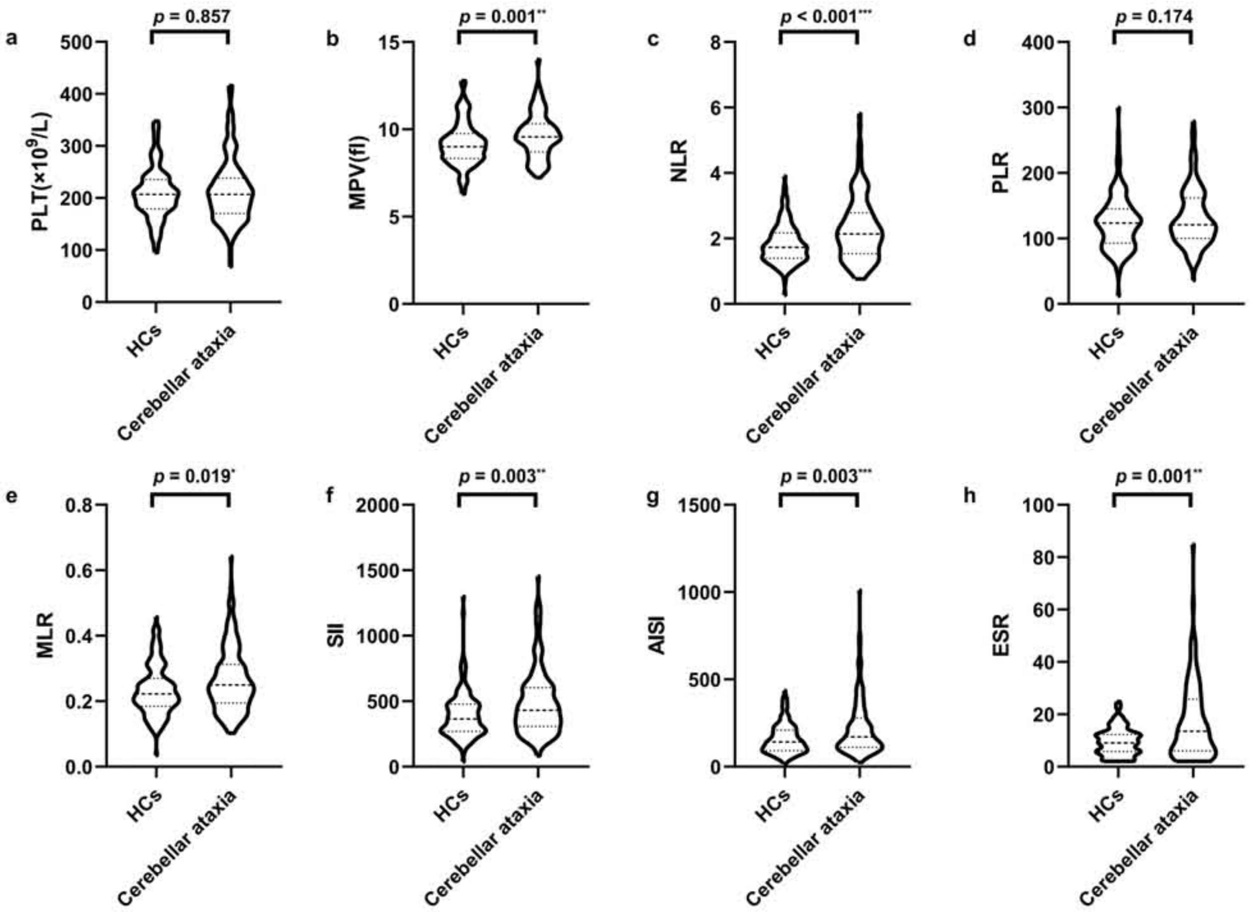

Fig. (S1). Comparisons of peripheral inflammatory in cerebellar ataxia patients and HCs. Variables were compared between two groups by independent sample t-test for normally distributed data or Mann-Whitney U test for abnormally distributed data. **Abbreviations**: PLT, platelet. MPV, mean platelet volume. NLR, neutrophils-to-lymphocyte ratio. PLR, platelet-to-lymphocytes ratio. MLR, monocytes-to-lymphocyte ratio. SII, systemic inflammation index. AISI, aggregate index of systemic inflammation. ESR, erythrocyte sedimentation rate. * p<0.05; ** p<0.01; *** p<0.001.

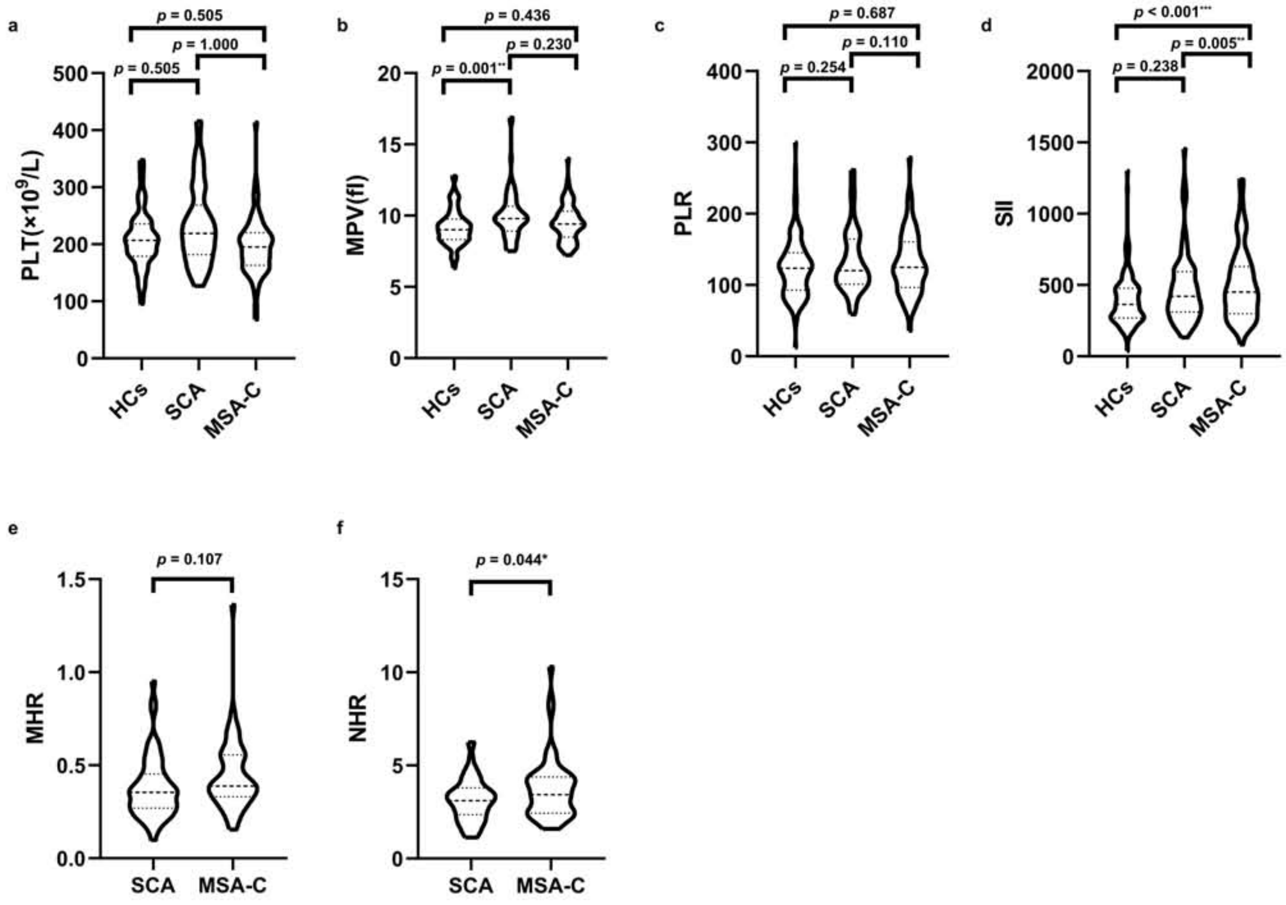

Fig. (S2). Comparisons of partial peripheral inflammatory in HCs, SCA and MSA-C subjects. Variables were compared among three groups by one-way ANOVA for normally distributed data or Kruskal-Wallis test for abnormally distributed data. *P* values of the posterior comparisons were adjusted by Bonferroni correction. Variables were compared between two groups by Mann-Whitney U test. **Abbreviations**: PLT, platelet. MPV, mean platelet volume. PLR, platelet-to-lymphocytes ratio. SII, systemic inflammation index. MHR, monocyte to high-density lipoprotein ratio. NHR, neutrophil to high-density lipoprotein ratio. * $p<0.05$; ** $p<0.01$; *** $p<0.001$ (Bonferroni corrected as appropriate)

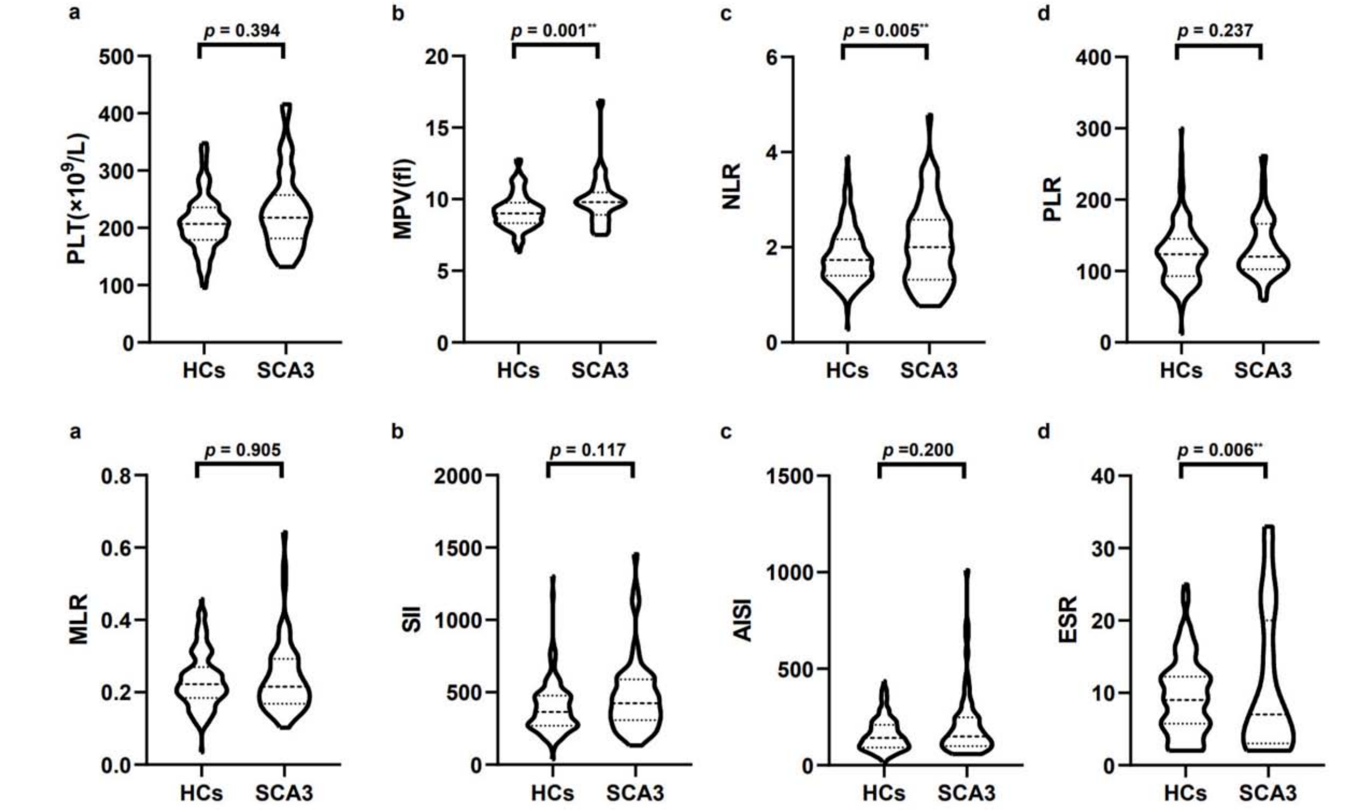

Fig. (S3). Comparisons of partial peripheral inflammatory between HCs and SCA3 subjects. Variables were compared between two groups by independent sample t-test for normally distributed data or Mann-Whitney U test for abnormally distributed data.

Abbreviations: PLT, platelet. MPV, mean platelet volume. NLR, neutrophils-to-lymphocyte ratio. PLR, platelet-to-lymphocytes ratio. MLR, monocytes-to-lymphocyte ratio. SII, systemic inflammation index. AISI, aggregate index of systemic inflammation. ESR, erythrocyte sedimentation rate. * $p<0.05$; ** $p<0.01$; *** $p<0.001$

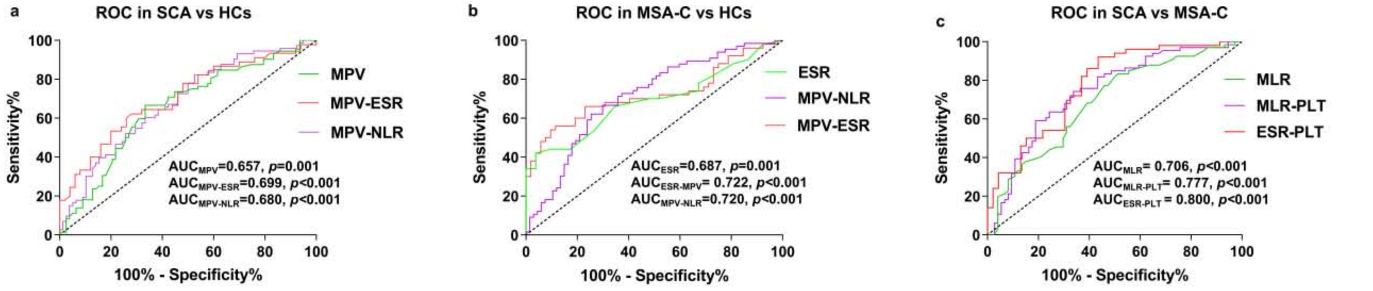

Fig. (S4). ROC curves to evaluate the utility of peripheral inflammatory profiles for differentiating SCAs, MSA-C and HCs groups. Sex-and-age matched healthy controls were used to exclude the influence of ages. a. ROC curves for distinguishing SCAs patients and HCs. b. ROC curves for distinguishing MSA-C patients and HCs. c. ROC curves for distinguishing SCAs and MSA-C patients. MPV, mean platelet volume. **Abbreviations**: ESR, erythrocyte sedimentation rate. NLR, neutrophils-to-lymphocyte ratio. MLR, monocytes-to-lymphocyte ratio.

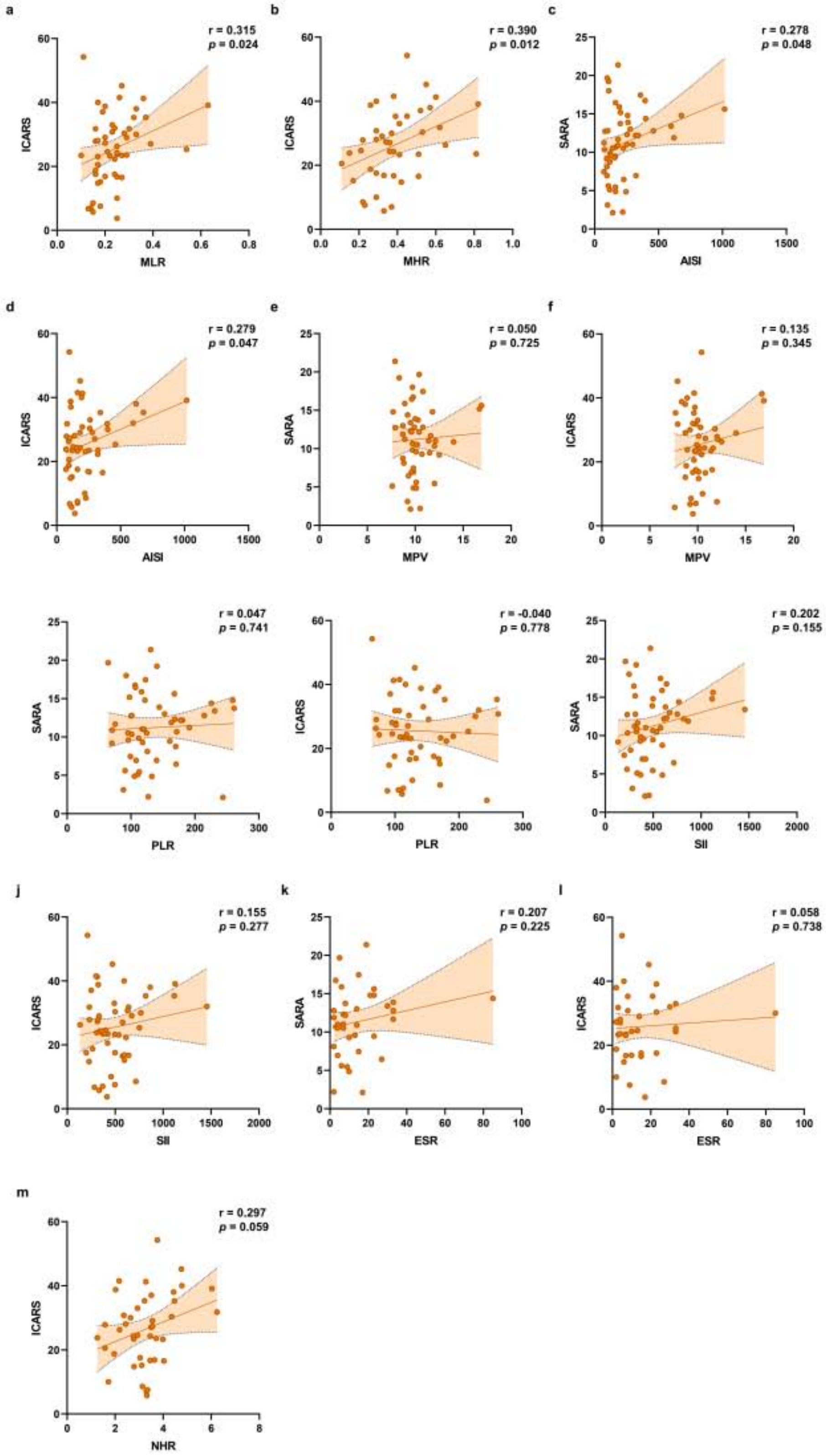

Fig. (S5). Correlation analysis between partial inflammatory markers and the severity of ataxia in SCA patients was presented. R values and *p* values calculated using Pearson correlation or Spearman correlation coefficient analysis are indicated on each dot plot. After Bonferroni correction, adjusted *p* values were non-significant. **Abbreviations**: MLR, monocytes-to-lymphocyte ratio. MHR, monocyte to high-density lipoprotein ratio. AISI, aggregate index of systemic inflammation. MPV, ICARS, the International Cooperative Ataxia Rating Scale. SARA, the Scale for the Assessment and Rating of Ataxia.

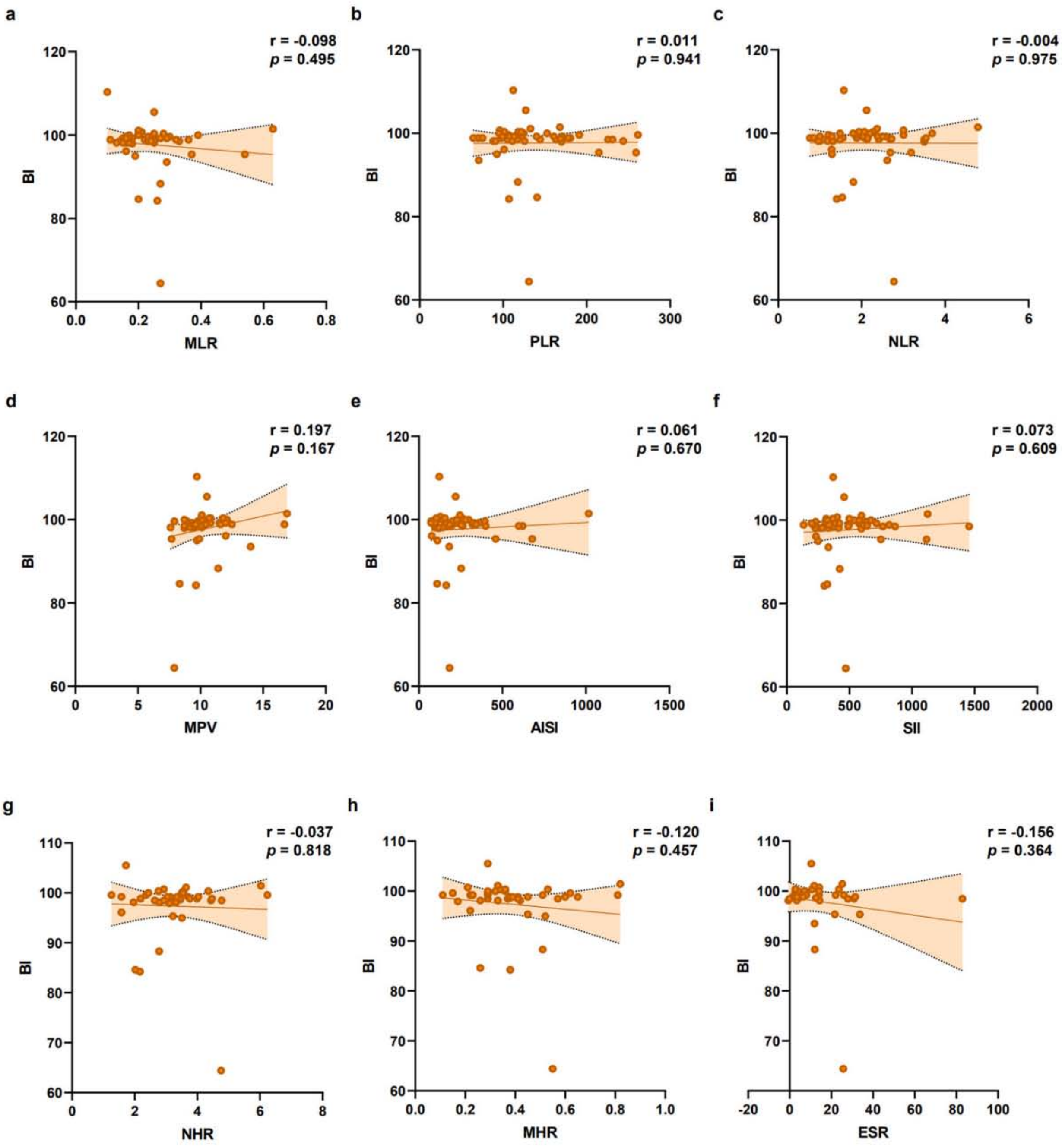

Fig. (S6). Correlation analysis between inflammatory markers and the Barthel Index (BI) in SCA patients was presented. R values and *p* values calculated using Pearson correlation or Spearman correlation coefficient analysis are indicated on each dot plot. After Bonferroni correction, adjusted *p* values were non-significant. **Abbreviations**: MPV, mean platelet volume. NLR, neutrophils-to-lymphocyte ratio. PLR, platelet-to-lymphocytes ratio. MLR, monocytes-to-lymphocyte ratio. SII, systemic inflammation index. AISI, aggregate index of systemic inflammation. MHR, monocyte to high-density lipoprotein ratio. NHR, neutrophil to high-density lipoprotein ratio. ESR, erythrocyte sedimentation rate. BI, Barthel Index.

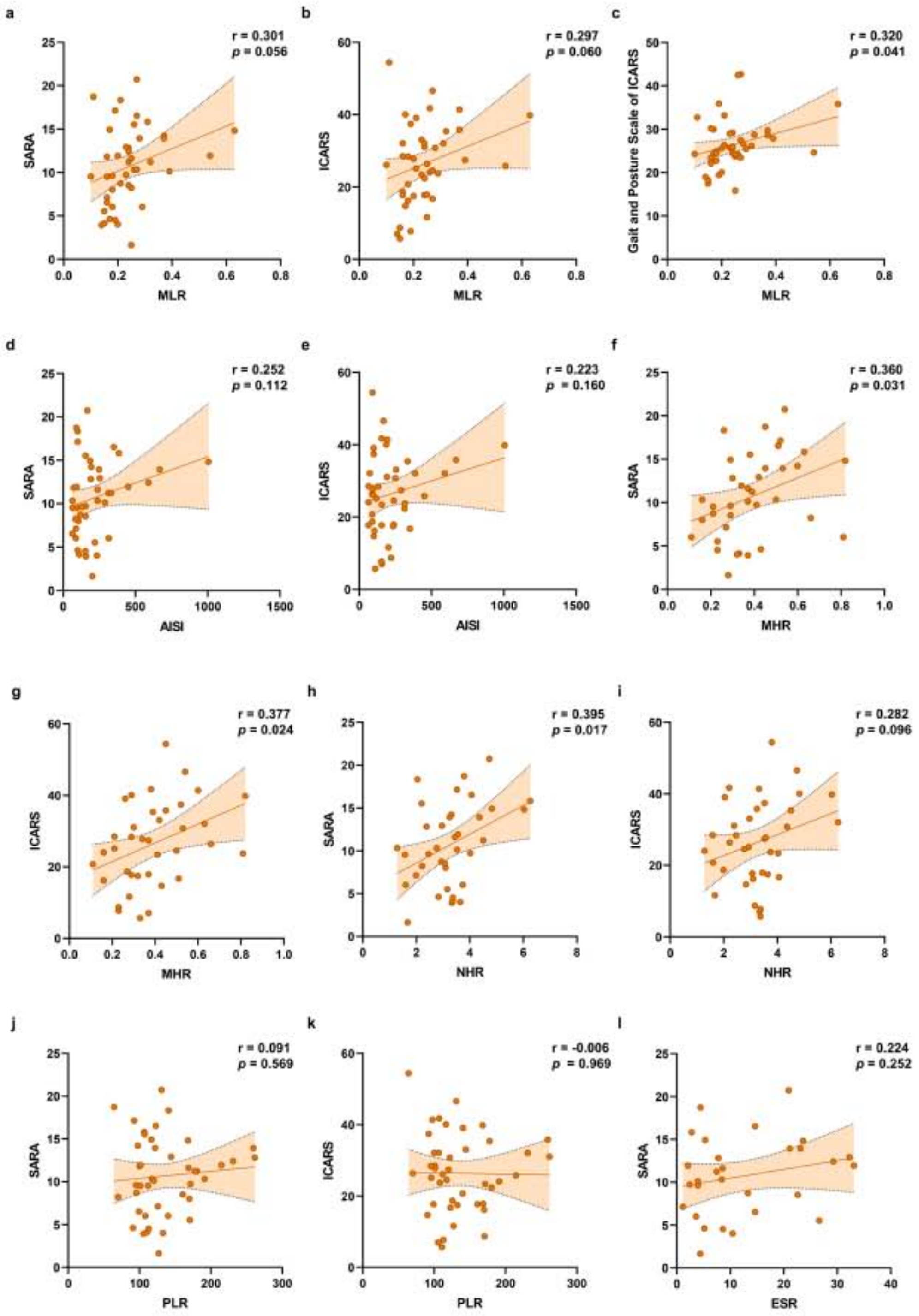

Fig. (S7). Correlation analysis between partial inflammatory markers and the severity of ataxia in SCA3 patients was presented. R values and *p* values calculated using Pearson correlation or Spearman correlation coefficient analysis are indicated on each dot plot. After Bonferroni correction, adjusted *p* values were non-significant. **Abbreviations**: MLR, monocytes-to-lymphocyte ratio. AISI, aggregate index of systemic inflammation. MHR, monocyte to high-density lipoprotein ratio. NHR, neutrophil to high-density lipoprotein ratio. PLR, platelet-to-lymphocytes ratio.SARA, the Scale for the Assessment and Rating of Ataxia. ICARS, the International Cooperative Ataxia Rating Scale.

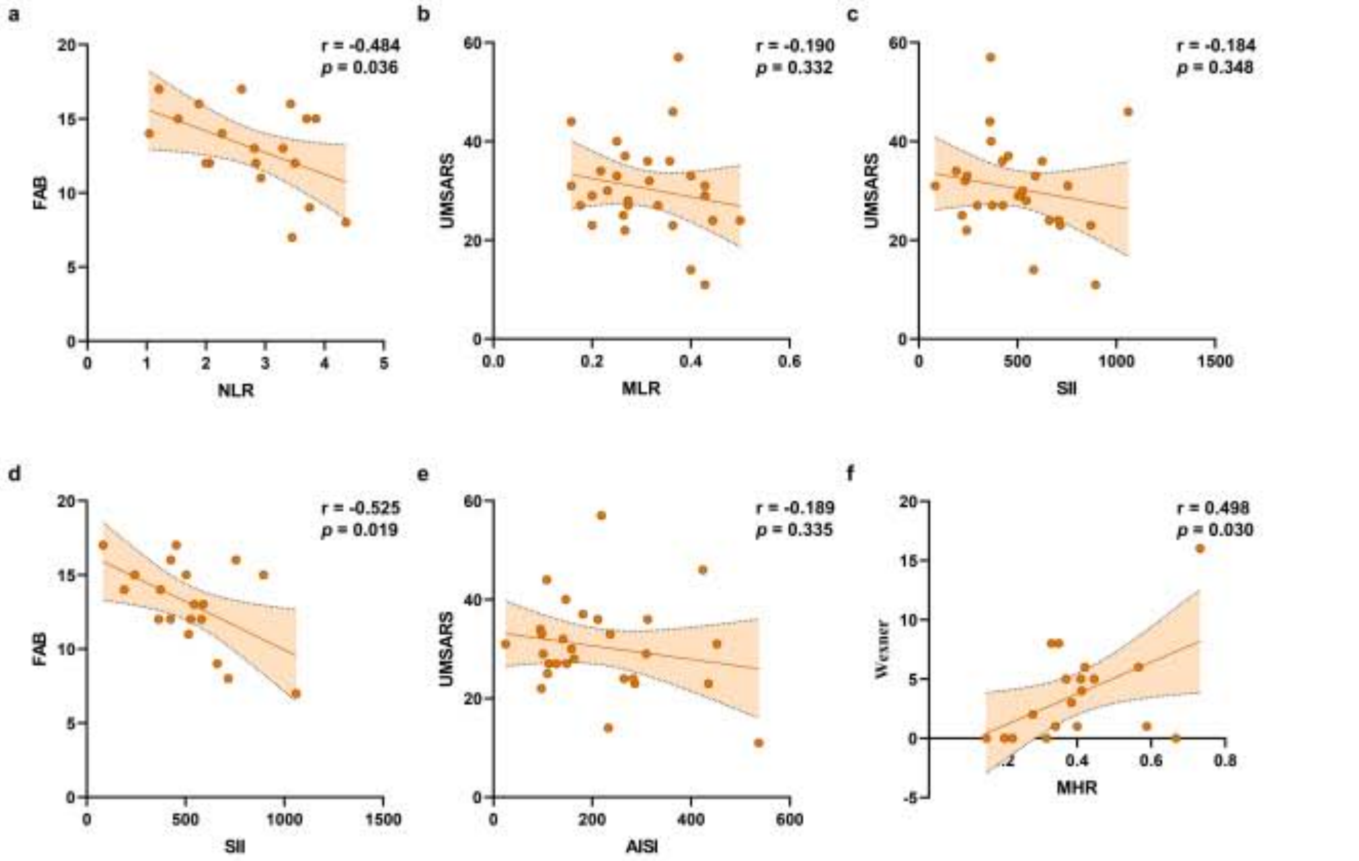

Fig. (S8). Correlation analysis between partial inflammatory markers and the severity of ataxia in MSA-C patients was presented. R values and *p* values calculated using Pearson correlation or Spearman correlation coefficient analysis are indicated on each dot plot. After Bonferroni correction, adjusted *p* values were non-significant. **Abbreviations**: NLR, neutrophils-to-lymphocyte ratio. MLR, monocytes-to-lymphocyte ratio. SII, systemic inflammation index. AISI, aggregate index of systemic inflammation. MHR, monocyte to high-density lipoprotein ratio. UMSARS, the Unified Multiple System Atrophy Rating Scale. FAB, Frontal Assessment Battery.

Table S1. Comparison of inflammatory profiles of cerebellar ataxia and control groups.

	Control	Cerebellar Ataxia	Value	Sig.
	n=145	n=140		
Sex			0.53	0.467
Male	89	80		
Female	56	60		
Average Age	47.00 (20.00)	53.36 (7.63)	0.236	0.627
PLT (×10^9/L)	207.00 (57.00)	207.00 (57.00)	-0.18	0.857
MPV (fl)	9.01 (1.42)	9.57 (1.61)	-3.441	0.001**
NLR	1.733 (0.767)	2.134 (1.245)	-4.014	0.000***
PLR	122.72 (39.37)	120.83 (61.65)	-1.36	0.174
MLR	0.222 (0.085)	0.250 (0.117)	-2.345	0.019*
SII	364.50 (208.764)	427.36 (291.38)	-2.985	0.003**
AISI	141.47 (117.77)	169.29 (158.00)	-2.98	0.003**
ESR[a](mm/h)	9.00 (7.00)	13.00 (19.00)	-3.202	0.001**

Note: Chi-square tests were used to determine the difference in sex composition between the two groups. Mann-Whitney U test was used for comparison of inflammatory profiles between the two groups. a. 102 controls and 96 cerebellar ataxia patients underwent ESR.

PLT, platelet. MPV, mean platelet volume. NLR, neutrophils-to-lymphocyte ratio. PLR, platelet-to-lymphocytes ratio. MLR, monocytes-to-lymphocyte ratio. SII, systemic inflammation index. AISI, aggregate index of systemic inflammation. ESR, erythrocyte sedimentation rate.

* p<0.05; ** p<0.01; *** p<0.001.

Table S2. Comparison of inflammatory profiles between SCA3 and control groups.

Group	Control	SCA3	Value	Sig.
	n=145	n=64		
Sex			0.486	0.486
Male	89	36		
Female	56	28		
Average Age	47.00 (20.00)	34.50 (17.00)	-4.488	0.000***
PLT (×10^9/L)	207.00 (57.00)	217.50 (76.00)	0.729	0.394
MPV (fl)	9.01 (1.42)	9.76 (1.53)	-3.339	0.001**
NLR	1.733 (0.767)	2.058 (0.858)	-4.014	0.005**
PLR	122.72 (39.37)	120.00 (63.60)	-1.182	0.237
MLR	0.222 (0.085)	0.214 (0.119)	-2.345	0.905
SII	364.50 (208.764)	420.73 (159.41)	-1.566	0.117
AISI	141.47 (117.77)	149.14 (145.62)	-1.282	0.200
ESR[a] (mm/h)	9.00 (7.00)	7.00 (17.00)	7.940	0.006**

Note: PLT, platelet. MPV, mean platelet volume. NLR, neutrophils-to-lymphocyte ratio. PLR, platelet-to-lymphocytes ratio. MLR, monocytes-to-lymphocyte ratio. SII, systemic inflammation index. AISI, aggregate index of systemic inflammation. ESR, erythrocyte sedimentation rate.

a.102 HCs and 37 SCA3 patients underwent ESR.

*p<0.05. **p<0.01. ***p<0.001.

Table S3. Correlations between peripheral inflammatory factors.

		PLT	MPV	NLR	PLR	MLR	SII	AISI	NHR	MHR	ESR	HDL
PLT	r	1.000	-0.291**	-0.064	0.490**	-0.083	0.479**	0.474**	0.213*	0.223*	-0.010	-0.031
	Sig.	.	0.000	0.282	0.000	0.166	0.000	0.000	0.023	0.017	0.891	0.741
MPV	r	-0.291**	1.000	0.009	-0.292**	-0.016	-0.151*	-0.115	-0.133	-0.040	-0.007	0.094
	Sig.	0.000	.	0.886	0.000	0.792	0.011	0.054	0.161	0.676	0.919	0.322
NLR	r	-0.064	0.009	1.000	0.530**	0.630**	0.788**	0.662**	0.607**	0.184*	0.147*	-0.107
	Sig.	0.282	0.886	.	0.000	0.000	0.000	0.000	0.000	0.050	0.040	0.253
PLR	r	0.490**	-0.292**	0.530**	1.000	0.409**	0.753**	0.529**	0.305**	0.072	0.092	-0.054
	Sig.	0.000	0.000	0.000	.	0.000	0.000	0.000	0.001	0.445	0.202	0.567
MLR	r	-0.083	-0.016	0.630**	0.409**	1.000	0.469**	0.682**	0.429**	0.596**	0.111	-0.175
	Sig.	0.166	0.792	0.000	0.000	.	0.000	0.000	0.000	0.000	0.124	0.062
SII	r	0.479**	-0.151*	0.788**	0.753**	0.469**	1.000	0.860**	0.643**	0.260**	0.119	-0.141
	Sig.	0.000	0.011	0.000	0.000	0.000	.	0.000	0.000	0.006	0.099	0.135
AISI	r	0.474**	-0.115	0.662**	0.529**	0.682**	0.860**	1.000	0.672**	0.581**	0.162*	-0.134
	Sig.	0.000	0.054	0.000	0.000	0.000	0.000	.	0.000	0.000	0.023	0.154
NHR	r	0.213*	-0.133	0.607**	0.305**	0.429**	0.643**	0.672**	1.000	0.690**	0.136	-0.626**
	Sig.	0.023	0.161	0.000	0.001	0.000	0.000	0.000	.	0.000	0.207	0.000
MHR	r	0.223*	-0.040	0.184*	0.072	0.596**	0.260**	0.581**	0.690**	1.000	0.063	-0.623**
	Sig.	0.017	0.676	0.050	0.445	0.000	0.006	0.000	0.000	.	0.561	0.000
ESR	r	-0.010	-0.007	0.147*	0.092	0.111	0.119	0.162*	0.136	0.063	1.000	0.011
	Sig.	0.891	0.919	0.040	0.202	0.124	0.099	0.023	0.207	0.561	.	0.922
HDL	r	-0.031	0.094	-0.107	-0.054	-0.175	-0.141	-0.134	-0.626**	-0.623**	0.011	1.000
	Sig.	0.741	0.322	0.253	0.567	0.062	0.135	0.154	0.000	0.000	0.922	.

Note: Pearson or Spearman correlation test was used to assess correlation between two variables obeying normal distribution or not.

PLT, platelet. MPV, mean platelet volume. NLR, neutrophils-to-lymphocyte ratio. PLR, platelet-to-lymphocytes ratio. MLR, monocytes-to-lymphocyte ratio. SII, systemic inflammation index. AISI, aggregate index of systemic inflammation. MHR, monocyte to high-density lipoprotein ratio. NHR, neutrophil to high-density lipoprotein ratio. ESR, erythrocyte sedimentation rate.

*$p<0.05$. **$p<0.01$.

Table S4. Comparison of inflammatory profiles between the mild and moderate-to-severe group in SCA patients.

Group	Mild Group	Moderate-to-severe Group	Value	Sig.
	n=22	n=30		
Sex			0.422	0.516
Male	13	15		
Female	9	15		
Average Age	35.18 (13.43)	37.83 (11.76)	0.572	0.453
PLT(×10^9/L)	231.59 (62.85)	248.80 (80.51)	0.694	0.409
MPV (fl)	10.164 (1.369)	10.292 (2.135)	0.061	0.806
NLR	1.866 (1.251)	2.250 (1.186)	-1.811	0.076
PLR	124.14 (49.60)	145.49 (752.89)	2.516	0.119
MLR	0.195 (0.054)	0.272 (0.111)	9.134	0.004**
SII	402.05 (141.27)	563.47 (297.51)	5.538	0.023*
AISI	144.80 (63.09)	280.40 (212.05)	8.409	0.006**
NHR	2.861 (0.690)	3.538 (1.698)	-2.008	0.051
MHR	0.336 (0.167)	0.366 (0.208)	3.476	0.070
ESR[a] (mm/h)	10.38 (7.438)	18.14 (18.816)	2.424	0.128

Note: PLT, platelet. MPV, mean platelet volume. NLR, neutrophils-to-lymphocyte ratio. PLR, platelet-to-lymphocytes ratio. MLR, monocytes-to-lymphocyte ratio. SII, systemic inflammation index. AISI, aggregate index of systemic inflammation. MHR, monocyte to high-density lipoprotein ratio. NHR, neutrophil to high-density lipoprotein ratio. ESR, erythrocyte sedimentation rate.

a. 16 mild and 21 moderate-to-severe patients underwent ESR.

*p<0.05. **p<0.01. ***p<0.001.

Table S5. Comparison of inflammatory profiles between the mild and severe group in MSA-C patients.

Group	Mild Group	Severe Group	Value	Sig.
	n=14	n=14		
Sex			0.848	0.357
Male	12	10		
Female	2	4		
Average Age	53.36 (8.25)	55.82 (6.48)	0.773	0.387
PLT(×10^9/L)	176.50 (34.180)	191.00 (51.136)	-0.882	0.386
MPV(fl)	9.330 (1.015)	9.276 (1.183)	0.130	0.897
NLR	3.009 (0.955)	2.228 (0.844)	2.293	0.030*
PLR	141.23 (42.425)	129.78 (56.068)	0.610	0.547
MLR	0.325 (0.104)	0.292 (0.864)	0.925	0.363
SII	539.09 (215.71)	448.05 (253.64)	1.023	0.316
AISI	228.90 (134.28)	200.56 (123.29)	0.341	0.564
NHR	3.480 (1.199)	3.347 (1.115)	0.289	0.775
MHR	0.404 (0.155)	0.432 (0.154)	-0.446	0.660
ESR[a](mm/h)	12.91 (6.96)	27.17 (18.89)	-2.440	0.028*

PLT, platelet. MPV, mean platelet volume. NLR, neutrophils-to-lymphocyte ratio. PLR, platelet-to-lymphocytes ratio. MLR, monocytes-to-lymphocyte ratio. SII, systemic inflammation index. AISI, aggregate index of systemic inflammation. MHR, monocyte to high-density lipoprotein ratio. NHR, neutrophil to high-density lipoprotein ratio. ESR, erythrocyte sedimentation rate.

a. 11 mild and 12 severe patients underwent ESR.

*p<0.05. **p<0.01. ***p<0.001.
